# Release of delta-9-tetrahydrocannabinol from polyvinyl alcohol hydrogels and its safe interaction with human skin fibroblasts

**DOI:** 10.3389/fddev.2024.1303812

**Published:** 2024-01-31

**Authors:** Shujun Cui, Ze Zhang, Denis Rodrigue, François Béland, Mahmoud Rouabhia

**Affiliations:** ^1^ Groupe de Recherche en Écologie Buccale, Faculté de Médecine Dentaire Université Laval, Québec, QC, Canada; ^2^ Axe Médecine Régénératrice Centre de Recherche du CHU de Québec—Université Laval, Département de Chirurgie Faculté de Médecine, Université Laval, Québec, QC, Canada; ^3^ Chemical Engineering Department, Laval University, Québec, QC, Canada; ^4^ SiliCycle^®^ Inc 2500, Parc-Technologique Blvd, Québec, QC, Canada

**Keywords:** cannabis, THC, hydrogel, cell adhesion, skin, fibroblasts

## Abstract

This study aimed to design a THC-rich hydrogel to deliver cannabis derivatives topically. We developed hydrogels using polyvinyl alcohol (PVA) mixed with propylene glycol (PG), vegetable glycerin (VG), or both to facilitate the dissolution of delta-9-tetrahydrocannabinol (THC). The hydrogels showed a brown color, confirming the presence of the cannabinoid. They exhibit a porous structure and better mechanical properties than PVA alone. Indeed, the hydrogel containing PG, VG, or both showed elastic deformation behaviors with lower water content. FTIR analysis demonstrated the presence of THC with two specific peaks at 1,575 and 1,619 cm^−1^, confirming the presence of THC in the hydrogels. Human dermal fibroblast cultures onto the surface of all hydrogels confirmed the safety of the THC-rich hydrogel as the cell adhesion was comparable to the control (no THC). Furthermore, cells adhering to the hydrogels could proliferate, showing increased cell viability at 48 and 72 h, with a higher proliferation obtained with the THC-rich PVA-PG-VG hydrogels. Such cell behavior could be due to the release of the THC in the culture medium, as demonstrated by ultra-high performance liquid chromatography (UPLC), showing the presence of THC in the culture medium, ranging from 203 to 290 μg after 24 h of incubation of the hydrogels containing PG and VG or both. In comparison, the released THC from the PVA hydrogel was higher, reaching 852 μg. It is interesting to note that the THC release at 24, 48, and 72 h was slower with the hydrogels containing PG, VG, and both, compared to PVA alone. Overall, the present study has designed safe THC-rich PVA-PG-VG hydrogels as a functional delivery system for the topical use of cannabinoids to control tissue diseases, such as inflammation.

## 1 Introduction

Cannabis sativa contains more than 500 constituents, one-fifth of which are phytocannabinoids, a group of C21 or C22 (for the carboxylated forms) terpenophenolic compounds. Over 100 phytocannabinoids have been identified (Stefkov et al., 2022). The predominant compounds are delta-9-tetrahydrocannabinol (THC), cannabidiol (CBD), and cannabinol (CBN), followed by cannabigerol (CBG) and cannabichromene (CBC) (Rong et al., 2017; Stefkov et al., 2022). The therapeutic benefits of cannabis have been ascribed to a diverse set of bioactive chemicals, such as cannabinoids, terpenes, and flavonoids, with cannabinoids being the most well-known. Although the pharmacology of most cannabinoids is unknown, THC and CBD have been studied as key contributors to the therapeutic effects of cannabis (Afrin et al., 2020; Śledziński et al., 2020).

Cannabis has long been used to relieve symptoms such as pain, fever, anxiety, and diarrhea in the context of numerous diseases (García-Gutiérrez et al., 2020). Furthermore, cannabis products were reported to reduce inflammatory diseases (Dalavaye et al., 2022). Over the past decades, it has been demonstrated that cannabinoids have anti-inflammatory effects, as ascertained by the decrease in the secretion of inflammatory mediators (Thapa et al., 2018; Preteroti et al., 2022). The human body is subjected to various conditions (stress, autocrine/endocrine changes, exposure to exogenous stimuli, etc.) leading to organ and tissue inflammatory disorders, such as those in the skin and the oral cavity. Such tissue inflammation could be controlled using cannabis products (Thapa et al., 2018; Preteroti et al., 2022).

Multiple routes can be used to administer cannabis products as medication (Stella et al., 2021; Parad et al., 2022). The routes include administration through food/beverages, orally as tablets or oil, transmucosal as a spray, and transdermal as a cream; the latter provides an easy way to deliver medical cannabis products to control local tissue inflammation. For a better-controlled delivery of cannabis molecules, a cannabis-rich polymer scaffold could be appropriate.

Polymeric drug delivery systems have enhanced stability and controlled the release of cannabis and cannabinoids. Poly (lactic-co-glycolic acid) (PLGA) was used for drug encapsulation due to its biocompatibility and biodegradability (Liechty et al., 2010; Bruni et al., 2018). CB-loaded nanoparticles coated with a range of agents (chitosan, vitamin E, and lecithin) were suggested for oral administration, creating nanoparticles ranging from 250 to 350 nm in size with good entrapment efficiency (Durán-Lobato et al., 2014). Cannabinoid-loaded PLGA nanoparticles were used in an ovarian cancer model showing decreased tumor growth (Fraguas-Sánchez et al., 2020). Although cannabis and cannabinoids can be encapsulated for clinical use, concerns about their bioavailability lead to the development of various cannabis formulations to improve bioavailability. Transmucosal and dermal delivery of cannabis and cannabinoid products have been suggested to solve certain limitations encountered with the oral administration of cannabis products (Laux et al., 2019). Several approaches have been used to deliver cannabis and cannabinoids through the skin (Stinchcomb et al., 2004). These include cannabinoid-rich patches (Paudel et al., 2010), cannabinoid-rich propylene glycol-hydroxyethyl cellulose gel, and cannabinoid-rich diethylene glycol monoethyl ether gel formulation (Osborne and Musakhanian, 2018). Cannabis and cannabis delivery to the skin can also be achieved through polymeric hydrogels, and the hydrogels can be generated using polyvinyl alcohol (PVA). This polymer has been used for numerous biomedical and pharmaceutical applications (Phan et al., 2019; Gonçalves et al., 2022; Mirzaeei et al., 2022). PVA hydrogels offer a non-toxic, non-carcinogenic, and bio-adhesive delivery system. PVA gels have been used for contact lenses, drug delivery applications, and more (Phan et al., 2019; Gonçalves et al., 2022; Mirzaeei et al., 2022). The presence of glycerol can improve the mechanical properties of PVA hydrogels (Delavari et al., 2022; Fu et al., 2022). Thus, PVA can be a good candidate for designing THC-rich hydrogels. This study aims to produce THC-rich PVA hydrogels and evaluate the presence and release of THC from such hydrogels.

The constructed hydrogels were able to retain the THC product. The THC was not released completely in a water-based solution, while it was released entirely in methanol. Since THC is lipophilic, our study suggests the possible delivery of THC when in contact with the tissues, including skin and oral mucosa, as the cells have lipid-rich membranes. Our THC-rich PVA hydrogels may have the potential as a drug carrier for the treatment of tissue inflammation.

## 2 Materials and methods

### 2.1 Reagents

Poly(vinyl alcohol) (PVA, #34584, Sigma-Aldrich, Oakville, ON, Canada). Sodium dodecylbenzenesulfonate (DBS, #289957, Sigma-Aldrich). Propylene glycol (PG, Sigma-Aldrich). Vegetable glycerine (VG, Sigma-Aldrich). High-purity grade THC was extracted from cannabis biomass and purified by SiliCycle Inc. (Quebec, Canada). The extracts of THC are derived from dried hemp biomass of Canadian cannabis plants. The hemp biomass was irradiated before extraction process. The biomass size was between 0.25 and 3 inches and was grinded with a 0.25 inches screen, before THC extraction.

### 2.2 Preparation of hydrogels

To generate the hydrogel, 10% (w/v) PVA and 5% (w/v) DBS (emulsifier) were added to distilled water to generate a PVA/DBS solution. The mixture was heated to 90°C and stirred vigorously until a homogeneous solution was formed. PG, VG, and PG-VG were added separately to the PVA solution and stirred vigorously until forming a homogeneous mixture. The PG, VG and PG-VG were added at 50% of the final volume of the PVA solution. The PVA-PG, PVA-VG, and PVA-PG-VG solutions were cooled down, then 0.5 mL of each of them was mixed with 15 μL of THC (1.8 mg), stirred to homogenize all chemicals, and transferred to wells of a 12-well tissue culture plate. The plates were subjected to 8 cycles of freeze-thaw. Each cycle included incubation at −80°C for 1 h to freeze the solution; then, the plates were moved to room temperature to thaw the solution (Wang et al., 2022). The designed hydrogels were then used for subsequent experiments. The PG and VG were added to improve the hydrogels’ mechanical properties and facilitate the solubility of the THC as a lipophilic product (Kim et al., 2015). We used such amounts of PG and VG as they allow for better dissolution of the THC oil in the PVA solution and contribute to a better distribution of the THC in the hydrogel.

### 2.3 Morphological structure of the hydrogels

Following the freeze-thaw cycles, the different solutions were examined manually to verify the formation of hydrogels. They were also inspected visually to record the color, especially for those solutions supplemented with THC. Obtained hydrogels were dried for 24 h at 37°C to evaluate whether the drying conditions led to hydrogel shrinking. Following such drying conditions, the obtained membranes were incubated for 60 and 120 min in saline to assess the possibility of rehydrating them. Freeze-dried hydrogels were also subjected to scanning electron microscopy (SEM, model JSM-6360LV, JEOL, Tokyo, Japan) analysis. We used an accelerating voltage of 10 kV to observe the microstructures of the membranes. The samples were snap frozen in liquid nitrogen, fractured, and sputter-coated with gold in a sputter coater (Fison Instruments, Polaron SC500, Uckfield, United Kingdom). Photomicrographs of various magnifications were taken from the representative parts of the surface and cross sections of 3 independent samples of each condition. The pore sizes were measured using the software ImageJ^®^ by measuring 20 pores in each image.

### 2.4 Swelling property of the hydrogels

The hydrogels were dried at room temperature and used to determine the swelling degree. Dried hydrogels were weighed and then placed in phosphate-buffered saline (PBS) for 24 h for rehydration. After this incubation, the weights of the hydrogels were determined. Finally, the swelling ratio was obtained using the following equation:

$$\text{Swelling ratio }\left(\text{wt}.\;\%\right)=100\times (\text{Weight of hydrated gel }‐\text{ Weight of dried gel})\;/\text{ weight of dried gel}.$$

### 2.5 Water content evaluation

Water content of the hydrogels was determined gravimetrically. Wet weight (W_w_) of water-saturated hydrogels was measured after wiping off their surface moisture with a lens cleaning paper. The samples were then dried (25°C, 50% humidity) until constant weight, and the dry weight (W_d_) was recorded. As previously reported (Williams et al., 2013), the water content was calculated according to the following formula:

$$\text{water content }\left(\%\right)=100\%\times \left(W_{w}‐W_{d}\right)/\;W_{w}$$

Measurements were performed on six samples for each type of hydrogel, and the water content results were expressed as the mean ± standard deviation.

### 2.6 Thermogravimetric analysis (TGA)

To further characterize the THC-rich hydrogels, we used a thermogravimetric analyzer TGA/SDTA 851e (Mettler-Toledo, Mississauga, Ontario, Canada). The different hydrogels containing or not THC were dried to constant weight before analyses. The TGA was run at a heating rate of 10°C/min from 25°C to 800°C in the atmosphere of nitrogen flowing at a rate of 20 mL/min, as we reported previously (Roohi et al., 2022). At least two specimens were tested for each hydrogel.

### 2.7 Mechanical properties of the hydrogels

Tensile tests were carried out to evaluate the mechanical properties of the hydrogels. Dog-bone samples were cut with the dimensions of 6.0 mm width at both ends and 2.0 mm × 10.0 mm in the middle, determined by a caliper (Fisherbrand™ Traceable™ Digital Calipers, United States), while the thickness was around 0.5 mm, determined by a thickness gauge under the pressure of 1.2 kPa (MTG-DX2, Rex Gauge Company, Buffalo Grove, IL, United States). The distance between both grips (gauge length) was fixed at 20 mm. To ensure constant and reproducible initial conditions, the samples were fixed with a torque wrench with a maximum torque of 35 cN.m. The tests were performed on a dynamic mechanical analysis (DMA) model RSA-3 (TA Instruments, United States). To improve adhesion between the samples and the grips and limit slippage, a mounting tape (3M Scotch^®^, United States) was used. The tests were conducted at a constant strain rate of 0.05 mm/s until failure, and the force was measured using a 350 g load cell. All the tests were done at room temperature with a relative humidity of 50%. Young’s modulus was obtained from the initial slope of the stress-strain curves (linear part), representing the materials’ rigidity. Because the pure PVA gels failed to break in the middle section of the specimens during the test, no mechanical test data of pure PVA gel were presented here. To compare the samples, *t*-Test (Microsoft Excel, United States) was applied for statistical analysis of the Young’s modulus, strain at break, and stress at break (tensile strength).

### 2.8 FTIR characterization

To determine the surface chemistry of the hydrogels, the hydrogels containing or not THC were washed with PBS for 24 h and then dried for 3 days to remove water. The dried hydrogels were subjected to Fourier transform infrared (FTIR) spectroscopy (Nicolet Instrument, Madison, United States) analyses in attenuated total reflectance (ATR) mode. For each specimen, 64 scans were performed between 400–4,000 cm^−1^ at a resolution of 4 cm^−1^. The spectra were analysed with the software provided by the instrument manufacturer, as we reported previously (Cui et al., 2021; Roohi et al., 2022).

### 2.9 Evaluation of the THC-rich PVA hydrogels’ safety

#### 2.9.1 Fibroblast adhesion

To evaluate the cell adhesion when in contact with the THC-rich PVA hydrogel, primary human skin fibroblasts (ATCC^®^ PCS-201-012™) were seeded (2 × 10^5) onto each type (PVA, PVA-PG, PVA-VG, or PVA-PG-VG) of hydrogel and cultured in Dulbecco’s modified Eagle’s medium (DMEM) with 10% fetal bovine serum, 100 U/mL penicillin G, 25 μg/mL streptomycin, and 0.5 μg/mL fungizone at 37°C in a 5% CO_2_ humid atmosphere for 24 h. At this time, the culture of each hydrogel was collected and centrifuged; the supernatant was aliquoted in three tubes and frozen at −80°C. The cells adhering to each hydrogel were fed fresh medium containing 10% of methyl thiazolyl tetrazolium salt (MTS) reagent (ab197010, Abcam, Cambridge, United Kingdom), and incubated for 3 h at 37°C in a dark atmosphere (Rouabhia et al., 2023). At the end of the incubation time, 200 μL, in triplicate, was placed in wells of a 96-well plate, and the absorbance was read at 499 nm using an X-Mark microplate spectrophotometer (Bio-Rad, Mississauga). The experiment was performed 3 times.

#### 2.9.2 Fibroblast proliferation

Human skin fibroblasts (10^5) were seeded on the surface of each hydrogel and cultured in Dulbecco’s modified Eagle’s medium (DME, Invitrogen Life Technologies, Burlington, ON, Canada) supplemented with 10% fetal bovine serum (FBS) (Gibco, Burlington, ON, Canada). The cultures were maintained for 24 h and 72 h at 37°C in a 5% CO_2_ humid atmosphere. At the end of each culture period, the medium was collected, centrifuged, aliquoted, and frozen at −80°C. The cells attached to the surface of the hydrogels were fed fresh medium supplemented with 10% of MTS reagent to evaluate the cell proliferation. Following incubation for 3 h, the absorbance was determined as described above. The experiment was performed 3 times. With a second set of experiments, we evaluated the fibroblast proliferation using trypan blue exclusion assay as we previously reported (Rouabhia et al., 2013). Fibroblasts were seeded onto each hydrogel and cultured for 48 or 72 h at 37°C in a humid atmosphere. At the end of each culture period, the cells were detached using a trypsin-EDTA solution. Live cells were resuspended in 1 mL of culture medium, then 100 µL of each cell suspension was mixed with 100 mL of trypan blue solution and subsequently incubated on ice for 5 min. The total number of live cells in each sample was determined by trypan blue exclusion. Viable cells refer to the cells that did not integrate the trypan blue. Results are reported as means ± SD of six assays.

#### 2.9.3 Collection of the rest of THC in the hydrogel after cell culture

Following the evaluation of the cell adhesion at 24 h and the cell proliferation at 48 h and 72 h, the hydrogels were incubated in 500 mL of methanol for 2 h under shaking. The collected solutions were used to measure the levels of THC representing what was left in the hydrogel after the cell culture periods.

#### 2.9.4 Measure the levels of THC in the cell culture supernatant

Supernatant collected in cell culture were used to measure the levels of THC. The measurements were performed by TransBioTech (Quebec, Qc, Canada). The following chemicals were used: methanol (MeOH), ethyl acetate, formic acid, acetonitrile, and isopropanol (Fisher Scientific). A THC standard curve was generated using delta-9-tetrahydrocannabinol (Millipore Sigma). To measure the levels of THC in our samples, 100 μL of each supernatant was mixed with 900 μL of dH_2_O plus 500 μL of ethyl acetate, mixed for 20 s, and then centrifuged for 5 min in a microfuge. The supernatant was transferred to a Reduced Surface Activity (RSA) vial, and the pellet was resuspended in 500 μL of ethyl acetate, mixed for 20 s, and centrifuged for 5 min. The supernatant was collected, added to the previous RSA tube, and evaporated at a dry atmosphere under azote at 30°C. After drying, 100 μL of MeOH/H_2_O (80%/20%) solution was added to the RSA tube, mixed then incubated in an ultrasound bath for 5 min. The solution was then injected in ultra-high performance liquid chromatography-MS/MS (UHPLC-MS/MS) (UPLC Waters Acquity XevoTQS). A standard curve was prepared using various concentrations (0.0 up to 500 ng/mL) of standard THC. All samples were analyzed using a Phenomenex Luna OMEGA C18, 2.1 × 150 mm, 1.6 µm reversed-phase column, at 40°C. The mobile phases and gradient were as follows: mobile phase A: 0.1% formic acid in dH_2_O; mobile phase B: 0.1% formic acid in acetonitrile. Flow rate: 0.3 mL/min. The programmed step gradient was 55% B over 2 min, 55%–100% B over 7 min, and 100%–55% B over 0.1 min. MS parameters in positive mode: desolvation temperature: 450°C; desolvation gas flow: 800 L/h; source temperature: 150°C; impactor: 2 kV; cone: 40 V, collision: 20 V. Collected values were used to determine the level of THC in each supernatant, based on the standard curve.

## 3 Results and discussion

### 3.1 Shape and structure of the hydrogels

**FIGURE 1:**
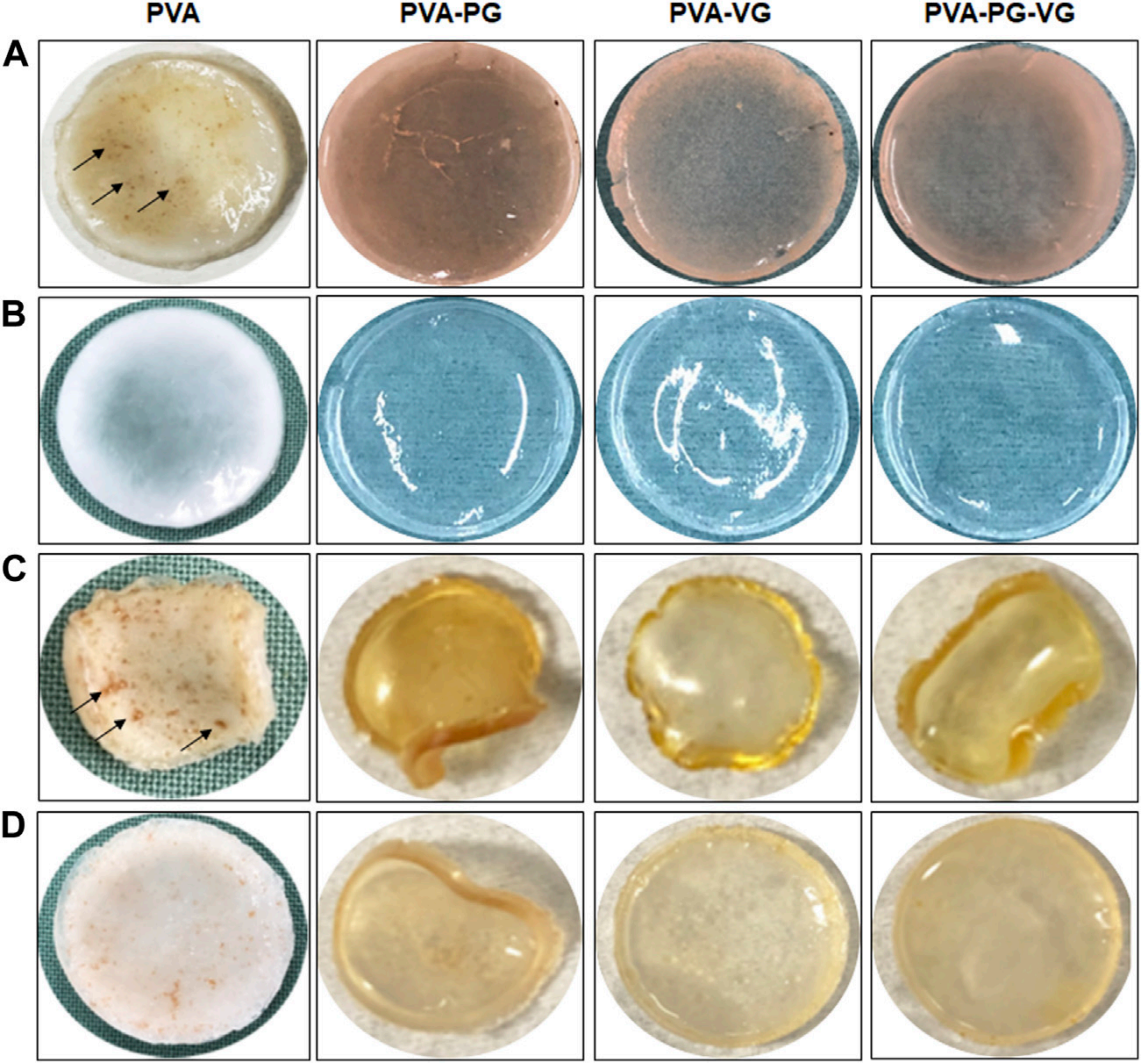
Production of THC-rich hydrogels. Hydrogels were produced using PVA-PG, PVA-VG, and PVA-PG-VG. The THC was added to the hydrogel before the 8 freeze-thaw cycles. The hydrogels were observed visually and photographed: **(A)** hydrogels immediately after the freeze-thaw cycles, **(B)** hydrogels without THC, **(C)** hydrogels after drying, and **(D)** hydrogels after rehydration in PBS.

**FIGURE 2:**
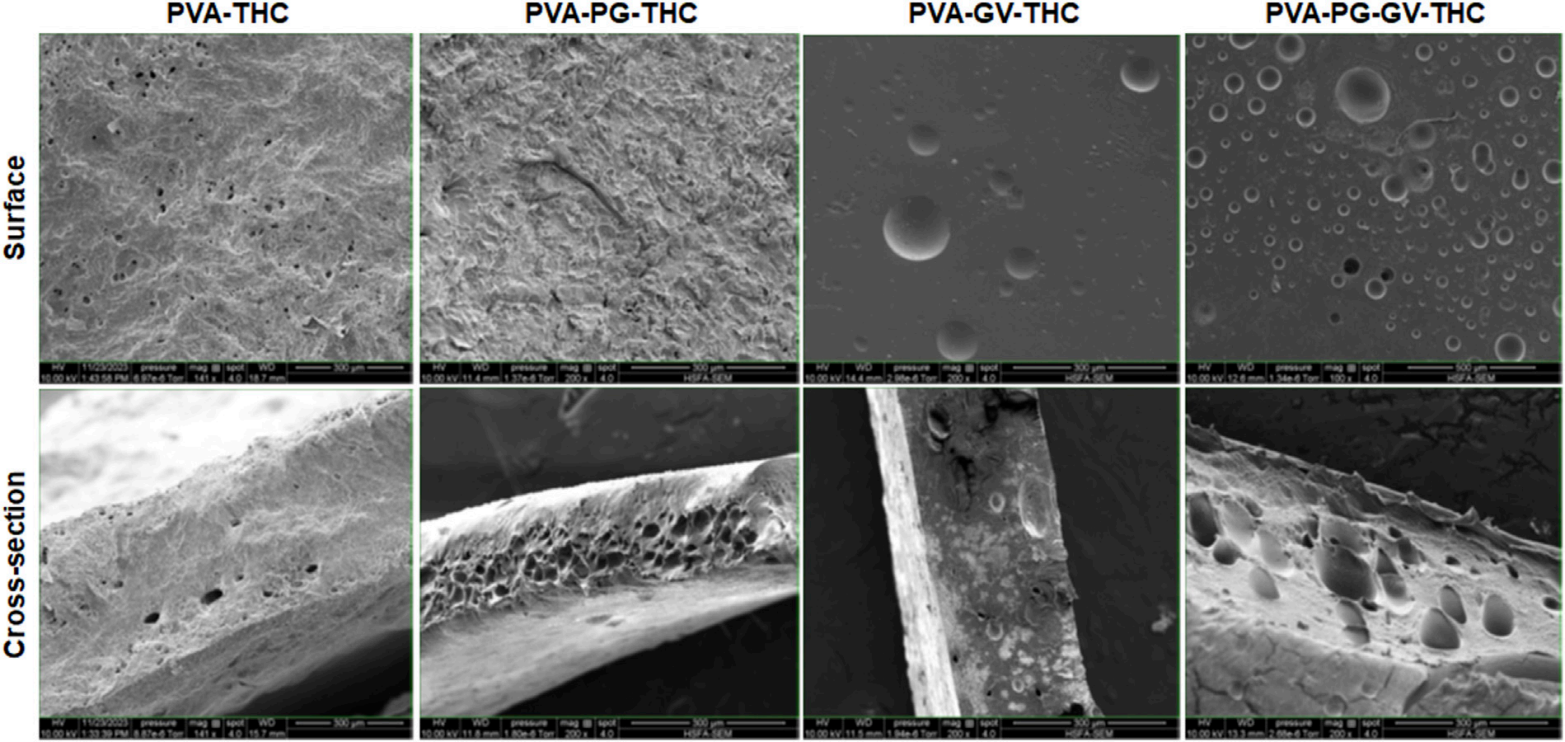
The structure of the hydrogels. Following their production, the PVA-PG, PVA-VG, and PVA-PG-VG hydrogels were analyzed by scanning electron microscopy (SEM). The analyses were performed on the surface and cross-sections of the hydrogels.

The PVA, PVA-PG, PVA-VG, and PVA-PG-VG solutions formed manipulable hydrogels after 8 freeze-thaw cycles (Figure 1A). These results follow those reported previously showing the formation of PVA hydrogels after physical (freeze-thaw) crosslinking (Mandal and Bostanian, 2000; Wang et al., 2022). There was no apparent difference in the shape or morphology of these hydrogels. The addition of THC did not inhibit the formation of the hydrogels (Figure 1A). The presence of PG and/or VG improved the mechanical properties (folding, twisting) of the hydrogels, making them easy to manipulate manually without any damage. Importantly, the presence of PG or VG significantly improved the dissolution and distribution of THC in the hydrogels. Indeed, in the absence of PG or VG, THC aggregated into brown spots of various sizes in the PVA hydrogels (Figure 1A, arrows), showing a distinct contrast as compared to those hydrogels containing PG or VG. The THC-rich PG or VG-hydrogels were uniformly brown, demonstrating the incorporation and uniform distribution of the cannabinoid (Figure 1A). In contrast, the THC-free hydrogels were white (PVA alone) or semi-transparent (PVA with PG or VG) (Figure 1B). All hydrogels maintained their structural stability at room temperature and 4°C. Drying them at 37°C for 24 h led to white-brown-colored membranes (Figure 1C) with slight shrinking. PVA hydrogel shrinking has been reported previously (Kudo et al., 2014). Incubation of the dried membranes in PBS for 60 min led to the re-formation of the hydrogel, with the recovery of the original size (Figure 1D). To further characterize the hydrogels, SEM analyses were performed to evaluate the surface and cross-section microstructures of the hydrogels. It should be noted that the PG, VG, and PG-VG were not analyzed as they could not form hydrogels for SEM observation. These results supported those reported previously, confirming the incapacity of PG, VG, and PG-VG to form manually manipulable hydrogels (de Brito et al., 2022). The SEM images of the hydrogels are presented in Figure 2. The surface of the hydrogels containing THC, especially the PVA-VG and PVA-PG-VG, showed the feature of micelles (Figure 2). The cross-section analyses showed the presence of a porous-like structure in all three different hydrogels (Figure 2). In the cross-sections, we can observe the presence of multiple pores with different pore sizes ranging from 26 ± 5 μm for the PVA-PG, 47 ± 22 μm for the PVA-VG, and 95 ± 26 μm for the PVA-PG-VG. These pores are attributed to oil droplets formed by THC in combination with PG, VG, and their mixture. However, the presence of THC alone only formed many much smaller pores on the surface and in the immediate subsurface layer, supporting that the large voids in other membranes were formed by PG or VG oil droplets (Figure 2). Such a feature was previously reported with PVA hydrogels containing xanthan gum microspheres (Bhattacharya et al., 2013).

### 3.2 Swelling degree of the hydrogels

As shown in Table 1, both PG and VG significantly affected the hydrogel swelling ratio, compared to the PVA alone. This ratio goes from 346% ± 5.1% with the PVA to 86.6% ± 1.1% with the PVA-PG, 42.9% ± 0.9% with the PVA-VG, and 79.0% ± 0.6% with the PVA-PG-VG. It is noted that the PVA-PG absorbed more water than the PVA-VG. However, in the PVA-PV-VG, the absorption level was comparable to that obtained with the PVA-PG. PVA chains are pushed together by water crystals to form a physically crosslinked network during repeated freeze-thaw processes (Wang et al., 2021), leaving void spaces within the polymer network (Alshangiti et al., 2023).

**TABLE 1:** Swelling ratio, water content and mechanical properties of the hydrogels.

	Hydrogels			
	PVA	PVA-PG	PVA-VG	PVA-PG-VG
Swelling ratio (%)	346.5 ± 5.1	86.8 ± 1.1	42.9 ± 0.9	79.0 ± 0.6
Water content (%)	83.6 ± 0.9	42.3 ± 0.9	27.8 ± 0.5	40.3 ± 0.9
Young’s modulus (kPa)		3.80 ± 0.30	5.78 ± 1.35	9.74 ± 1.35
Strain at break (%)		140.5 ± 9.1	155.9 ± 19.9	167.7 ± 34.8
Stress at break		1,164.9 ± 54.3	751.7 ± 166.1	1,560.8 ± 219.9

Consequently, owing to many hydroxyl groups and the stable 3D network, crosslinked PVA is highly hydrophilic and can absorb and hold many water molecules. When the dried PVA sponge was put into water, water penetrated the matrix by diffusion, capillary action, and hydrogen bonding, leading to a high volume of free and bound water integrated into the 3D network of PVA (Karoyo and Wilson, 2021). It was expected that the presence of hydrophobic PG or VG occupied some voids in the network and, at the same time, competed with water to bond with the hydroxyl groups on PVA chains, which hindered the migration of water from the environment into the polymer 3D network. The swelling ratio measures equilibrium when the hindering and absorbing forces, or the hydrophobic and hydrophilic effects, reach a balance. The high swelling ratio of PVA-PG could be explained by the fact that PG has fewer hydroxyl groups than VG. By assuming that the hydroxyl groups in PG and VG competed with water molecules to interact with the hydroxyl groups in PVA to form hydrogen bonds (Karoyo and Wilson, 2021; Wang et al., 2021; Alshangiti et al., 2023), the PVA molecules in PVA-PG gel would have more hydroxyl groups to interact with water, leading to a high swelling ratio. To confirm this explanation, we performed water content measurements (Table 1), showing that the PVA-PG and PVA-VG hydrogels contained up to approximately 40% of water as compared to the PVA alone (84%), while the PVA-VG hydrogel contained only about 28% of water. Retention of a large volume of water in the intermolecular space is related to the presence of hydrophilic functional groups in the network (Pohan et al., 2020). The data showed that the hydrogel containing PG has a higher water-locking capacity than that containing VG. The data showed that hydrogel with PG has a higher water locking capacity than with VG. Since the hydrophilic functional group of PG is less than VG, the most likely possibility is the microstructure of PVA-PG hydrogel is more favorable to trap water than PVA-VG hydrogel.

### 3.3 Thermogravimetric analysis (TGA)

**FIGURE 3:**
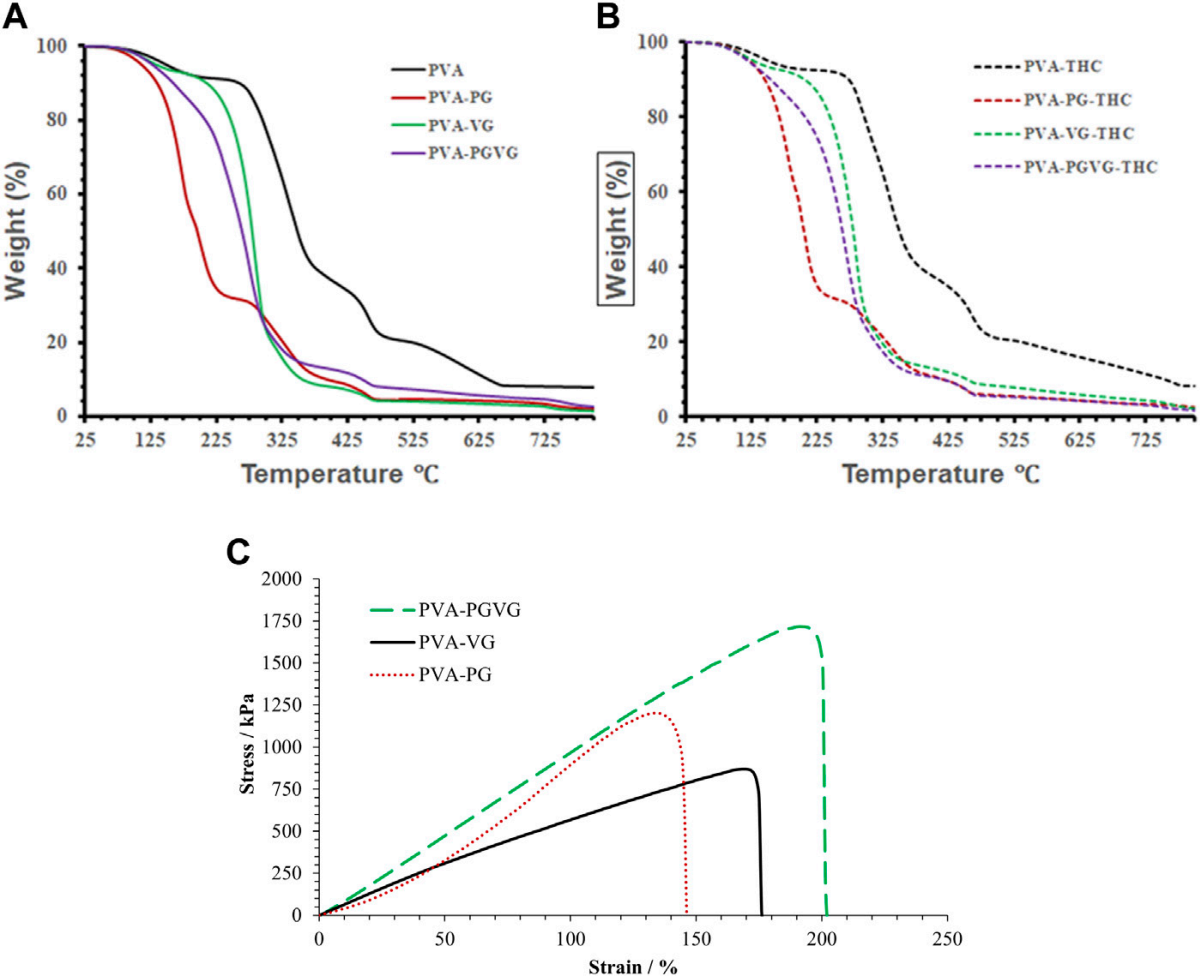
TGA analyses, and strain and stress curve of the hydrogels. **(A)** showed the TGA analyses THC free hydrogels. **(B)** shows the TGA analyses of the THC-rich hydrogels. **(C)** shows the strain and stress curve of the THC-rich hydrogels. PVA, poly (vinyl alcohol); PG, propylene glycol; VG, vegetable glycerin.

As presented in Figures 3A, B, the amount of THC showed no effect on the thermodegradation of the hydrogels. However, the presence of PG, VG, or both led to less stable thermostability that must be related to structural changes in the hydrogels. Indeed, the thermogravimetric curves of the PVA hydrogels free of PG and VG show four distinct temperature stages of weight loss (Figures 3A, B). The first stage ranges from 25°C to 250°C (accounting for 10% of the weight loss), where the weight loss is mainly from absorbed and hydrogen-bonded water. The second stage is from 250°C to 400°C (accounting for 55%), where the most significant weight loss happens, which is assigned to PVA decomposition (Awada and Daneault, 2015). The third stage is from 400°C to 500°C (accounting for 15%), which is related to the decomposition of DBS (Li et al., 2023). The fourth stage, from 500°C to 780°C (accounting for 10%), may be due to further decomposition of the substances that left about 10% of the residue at 800°C (Olvera Bernal et al., 2023).

For the PVA hydrogels containing PG or VG, the evaporation of oil partially overlapped with the decomposition of PVA, forming a major weight loss between 100 and 375°C. Among them, the PVA-PG recorded two distinct stages corresponding to the evaporation of PG starting around 120°C (Hong et al., 2021) and the decomposition of PVA in the second stage starting at about 225°C. For the PVA-VG, the evaporation of VG occurred between 120°C and 300°C (Almazrouei et al., 2019) and partially overlapped with PVA decomposition that ended around 375°C. The thermostability of the PVA-PG-VG is better than that of the PVA-PG but worse than that of the PVA-VG, as expected. The boiling points of pure PG and VG are 188 and 290°C, supporting that PG evaporated at a lower temperature than VG. Like PVA hydrogel, all other gels recorded the decomposition of DBS.

Generally, the decomposition temperatures shift towards lower temperatures when there is PG or VG in the hydrogel, probably because their presence loosens the microstructure of the hydrogels. Since the hydrogels are formed through the freeze–and-thaw-induced hydrogen bonds between PVA molecules, the presence of PG or VG and their interactions with PVA through hydrogen bonding should have prevented the chains of PVA from becoming connected. Consequently, the presence of VG and PG reduces ordered domains and crystal structures in PVA. It makes PVA largely amorphous, supported by the transparent appearance of the PG- and VG-containing hydrogels and the DSC results. Compared with crystalline PVA, amorphous PVA has a higher diffusivity favoring drug delivery.

### 3.4 Mechanical properties of the hydrogels

Figure 3C shows the stress-strain curves of the THC-rich hydrogels. The PVA-PG and PVA-PG-VG showed elastic deformation behaviors (straight line), which may indicate a high degree of crosslink. The PAV-VG, on the other hand, showed a positive change in slope during stretching and little or no yielding before failure, indicating some molecular interactions that strengthened the gel during deformation. Table 1 shows the data generated from the stress-strain curves. The difference in Yong’s modulus is significant between PVA-PG and PVA-PG-VG (*p*-value is 0.04); others are insignificant (*p*-values are more than 0.1). The difference in strain between different groups is insignificant (all the *p*-values are greater than 0.1). The difference in stress at break between PVA-PG and PVA-VG is marginally significant (*p*-value is 0.06), while others have no significant difference (*p*-values are greater than 0.1). As explained in Section 3.2, the PVA-PG gel would have more hydroxyl groups to interact with water during freeze-thaw processing, leading to more water crystals in the PVA-PG gel and, consequently, a higher degree of crosslink, leading to a higher breaking strength. However, this does not explain the low Young’s modulus of the PVA-PG and the properties of the gels when both PG and VG were incorporated into the same gel, i.e., the PVA-PG-VG. There must be other factors in play, for example, the plasticizing effect of the PG and VG and the size and number of the oil droplets in the gels (Lin et al., 2019; Chen et al., 2022). Compared with the water molecules absorbed through hydrogen bonding with PVA, PG and VG molecules are more mobile as they are less mixable with water. This mobility renders them as a lubricant to make the hydrogel network more elastic or stretchable. In contrast to the oil-containing hydrogels, as one can see from Table 1, the pure PVA hydrogel has a Young’s modulus comparable with other hydrogels; however, it is stiff and shows a low ability to deform (low strain). The oil droplets also provide spaces that are easily to form because oil is mobile.

### 3.5 FTIR analysis of the THC distribution

**FIGURE 4:**
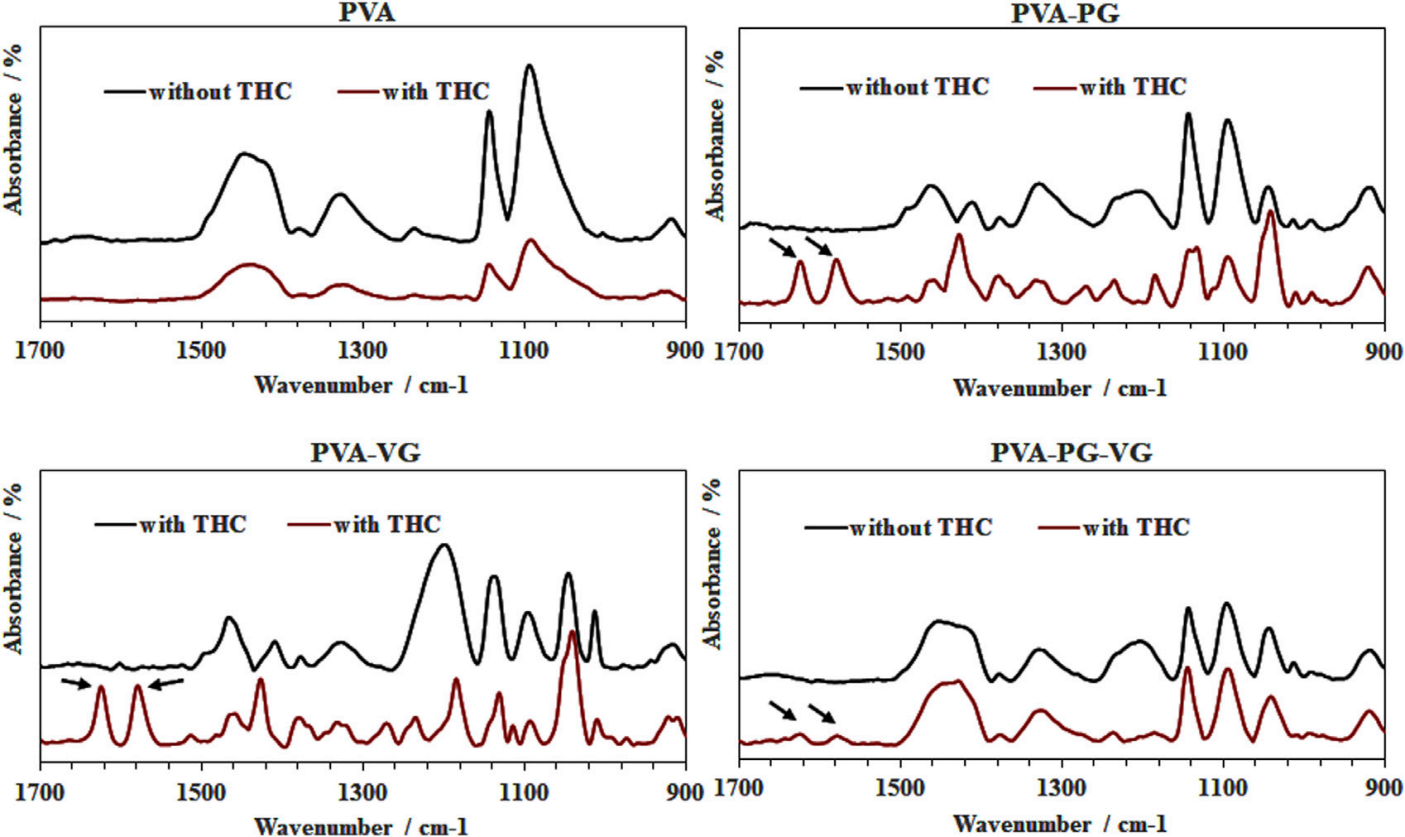
FTIR spectra confirming the presence of THC in the hydrogels. PVA, poly (vinyl alcohol); PG, propylene glycol; VG, vegetable glycerine. Arrows show THC specific picks.

The Figure 4 shows strong peaks at 1,575 and 1,619 cm^−1^ (arrows). The 1,619 cm^−1^ peak was attributed to C–C stretching in the benzene ring, ring deformation, and C–C–C deformation. The peak located at 1,575 cm^−1^ was associated with benzene ring stretching of C=C, as previously reported (Siano et al., 2018; Yao et al., 2023). As previously reported, these two peaks correspond to THC in our hydrogels (Cirrincione et al., 2021). It is interesting to note the strong THC signals in PVA-PG and PVA-VG samples. We also detected THC in the hydrogels containing PG and VG, but at low level, while no signal of THC in the PVA hydrogels. Considering the low sampling depth of ATR, which is normally less than 20 microns, these results demonstrated the distribution and availability of THC in the surface layer of the PG-, VG-rich hydrogels, suggesting the contribution of the PG and VG to the dissolution of the THC oil in the PVA hydrogel.

### 3.6 Skin fibroblasts adhered well to the THC-rich hydrogel

**FIGURE 5:**
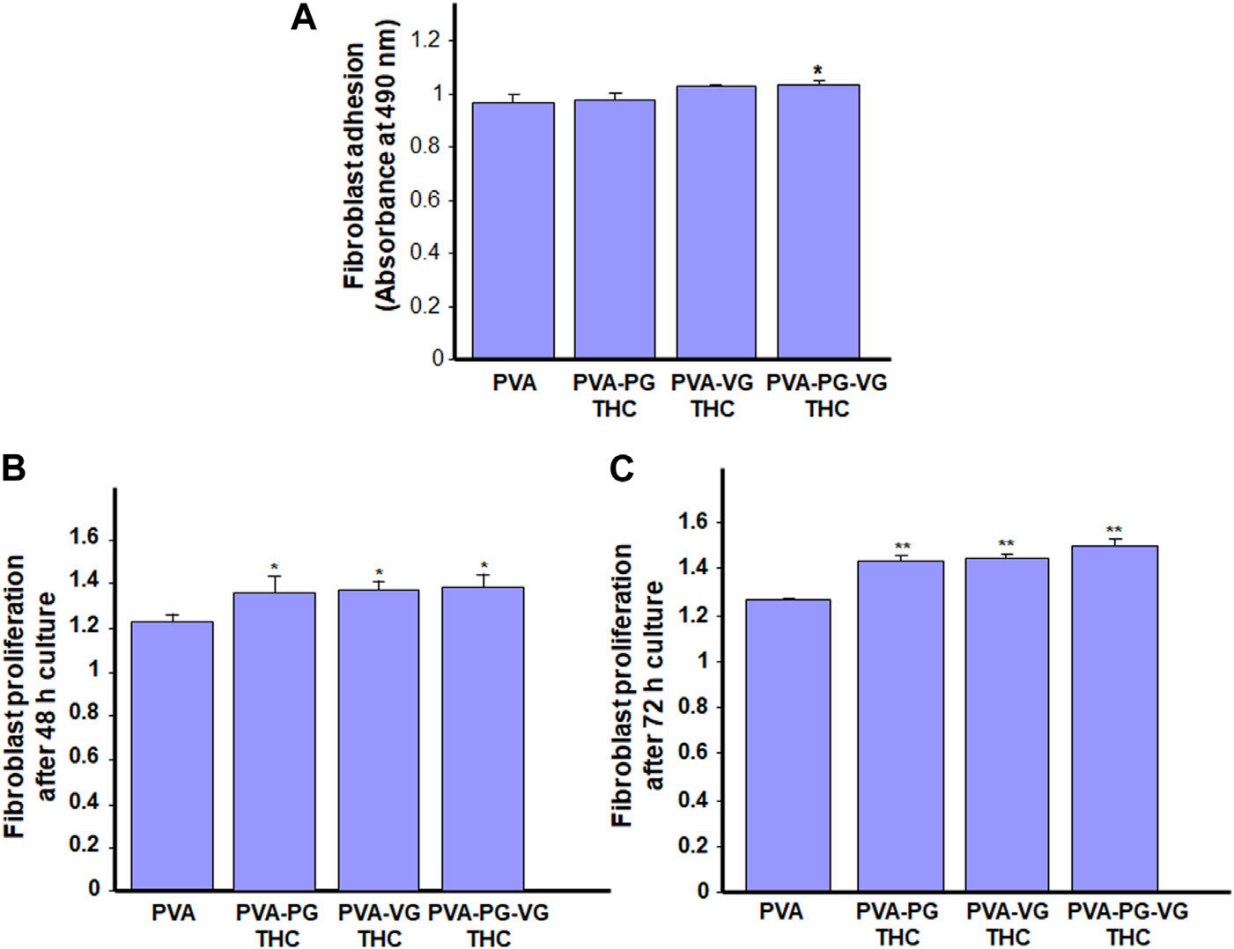
Safety of the THC-rich hydrogels. Human skin fibroblasts were used to evaluate the cell adhesion onto the hydrogels **(A)** and cell proliferation after 48 h **(B)** and 72 h **(C)**. Results are the means ± SD of 4 separate experiments (*n* = 4). Statistical significance was obtained by comparing the presence and the absence of THC in the hydrogels. * *p* < 0.05; **p < 0.01.

As the THC-rich hydrogels are intended for topical drug delivery through skin contact, they should be safe for human skin cells. With this study section, we evaluated the first key parameter of the safety of the hydrogel, i.e., skin fibroblast adhesion. As shown in Figure 5A, all gels show similar adhesion rates, or even better when the THC is incorporated. Indeed, the absorbance ranged from 0.97 ± 0.03 with the PVA, 0.98 ± 0.03 with the PVA-PG, 1.03 ± 0.01 with the PVA-VG, and 1.04 + 0.01 with the PVA-PG-VG. These results demonstrated the safety of the THC-rich PVA hydrogels. They also confirm findings previously reported involving PVA (Geskovski et al., 2021) and PVA mixed with active molecules such as chitosan (Eivazzadeh-Keihan et al., 2023) and collagen (Yu et al., 2021). Because the human skin fibroblasts adhered well to the THC-rich hydrogels, this may contribute to cell proliferation.

### 3.7 Skin fibroblasts proliferated once in culture on the THC-rich hydrogels

Human skin fibroblasts were seeded onto the THC-rich hydrogels, and their proliferation was assessed at 48 and 72 h. The results (Figure 5B) further confirmed the safety of these hydrogels as the fibroblasts proliferated on the PVA or THC-rich PVA hydrogels. The proliferation was even better in the presence of THC. Indeed, the absorbance at 48 h ranged from 1.22 ± 0.04 with the PVA alone to 1.26 ± 0.03 with the THC-rich PVA-PG, 1.29 ± 0.04 with the THC-rich PVA-VG, to 1.3 ± 0.03 with the THC-rich PVA-PG-VG hydrogels. Comparable results were obtained after 72 h cell culture (Figure 5C). To confirm these results; we performed viable cell counts using trypan blue exclusion assay. Table 2 showed that the number of live fibroblasts significantly increased after 48 and 72 h culture onto the hydrogels containing PG, VG or PG-VG compared to the PVA alone. These results are comparable to those generated with chitosan-rich PVA hydrogels (Huang et al., 2023) or sodium alginate (Alavarse et al., 2023), to list only a few. It is important to note that this is the first study designing cannabis-product-rich hydrogels and evaluating their safety. Cannabis products were already reported to improve dermal fibroblast regenerative properties (Gerasymchuk et al., 2022), supporting our study showing fibroblast proliferation after 48 and 72 h contact. Overall, this study section demonstrated the safety of our THC-rich hydrogels and suggested the possible use of these hydrogels as a THC drug delivery system. For this purpose, we measured the levels of THC in the cell culture medium.

**TABLE 2:** Cell proliferation after culture onto the THC-rich hydrogels (× 10^5).

(h)	PVA	PVA-PG	PVA-VG	PVA-PG-VG
48 h	10.8 ± 0.7	12.6 ± 0.5*	13 ± 0.3**	14 ± 0.6***
72 h	13.9 ± 0.3	15.5 ± 0.2*	16 ± 0.9**	17.6 ± 0.5***

*p < 0.05; **p < 0.01; ***p < 0.001 when comparing the PVA-PG, PVA-VG, and PVA-PG-VG, to PVA., cell proliferation was evaluated by trypan blue exclusion assay.

### 3.8 Release of THC in the cell culture medium

The supernatant collected from the 24, 48, and 72 h cell cultures was subjected to UPLC-MS/MS. The results are reported in Table 3, showing the amount of THC released in the culture medium. It is noted that the maximum release was obtained during the first 24 h. Indeed, the levels of THC ranged from 290 ± 65 μg in the supernatant of the PVA-PG, 235 ± 4 μg in the supernatant of the PVA-VG, and 203 ± 62 μg with the supernatant of the PVA-PG-VG hydrogels. We can note the low concentration of THC released from the hydrogels compared to the initial concentration (1,800 μg) included in each hydrogel. It demonstrated the slow release of THC from the hydrogel making them a good drug delivery system to administrate the THC for long period. Similar observations were made at 48 and 72 h. In the culture medium of 48 h, the levels of THC were 77 ± 4 μg with the PVA-PG, 76 ± 7 μg with the PVA-VG, and 79 ± 7 μg with the PVA-PV-VG hydrogels. At 72 h, there was also THC release in the culture medium, but at low levels (Table 3). It is interesting to note that the release of THC at 24, 48, and 72 h from the PVA hydrogels was much higher compared to those hydrogels containing PG, VG, and both, suggesting a burst release of about 50% of THC in the first 24 h and 33% in the next 2 days. Such a burst release can be well explained by the porous surface and subsurface of the PVA-THC membrane (Figure 2). On the other hand, a slow and better-controlled release of THC was achieved in the presence of PG and VG. Apparently, a slow and better-controlled release of THC was achieved in the presence of PG and VG. Such a slow release was probably because of the small oil droplets formed in the PG- and VG-containing hydrogels, as shown by SEM photos, which served as reservoirs of THC. The addition of the released THC amounts obtained at 24, 48, and 72 h did not match the amount added to the hydrogel. This suggests that the hydrogels still contain THC. For this purpose, we released all the THC still in the hydrogels after cell culture through the incubation in methanol. The UPLC-MS/MS measurement confirmed the presence of THC that was still trapped in the hydrogels. Table 3 showed that the levels of THC still in the hydrogels were 340 ± 70 μg in the PVA, 1,170 ± 12 μg in the PVA-PG, 1,407 ± 14 μg in the PVA-VG, and 1,315 ± 26 μg in the PVA-PV-VG hydrogels. The cumulative release of the THC from each type of hydrogel was almost the same as what was originally loaded in each hydrogel during their preparation, which was 1,800 μg. Indeed, with the PVA alone, the level of THC was 1,769 ± 24 μg; with the PVA-PG, the level of THC was 1,588 ± 88 μg; with the PVA-VG, the level was 1,751 ± 42 μg; and with the PVA-PG-VG, the THC level was 1,693 ± 97 μg. The loading efficiency was above 94% and almost equivalent for all the hydrogels except for the PVA-PG, which showed a slightly lower value of 88%. Overall, this study section demonstrated the possible topical use of PVA hydrogels to deliver THC continuously. To our knowledge, this is the first study elaborating on THC-rich hydrogel for topical applications. Our study supports those performed with THC-rich lipid nanoparticles, demonstrating their potential use for topical skin treatment (Kakkar et al., 2018; Saini et al., 2022).

**TABLE 3:** Release of THC in the culture medium from the different hydrogels determined by UPLC-MS/MS analysis.

	PVA (μg)	PVA-PG (μg)	PVA-VG (μg)	PVA-PG-VG (μg)
24 h	852 ± 45	290 ± 65	235 ± 4	203 ± 62
48 h	388 ± 55	77 ± 4	76 ± 7	79 ± 7
72 h	189 ± 10	51 ± 7	74 ± 17	45 ± 5
2 h in methanol	340 ± 70	1,170 ± 12	1,407 ± 14	1,315 ± 26
Cumulative release	1769 ± 24 (98%)	1,588 ± 88 (88%)	1751 ± 42 (97%)	1,693 ± 100 (94%)

## 4 Conclusion

We constructed a THC-rich PVA matrix. Adding PG, VG, and both to the PVA hydrogels contributes to the overall dissolution and distribution of the THC in the hydrogels compared to the THC in the PVA alone. Also, the presence of PG and VG improved the softness of the hydrogel. Indeed, the PVA-VG and PVA-PG-VG showed elastic deformation behaviors, suggesting a high degree of crosslink. The generated hydrogel showed high levels of THC, as shown by the strong peaks at 1,575 and 1,619 cm^−1^ following the FTIR analyses. The 1,619 cm^−1^ peak was attributed to C–C stretching in the benzene ring, ring deformation, and C–C–C deformation. The peak located at 1,575 cm^−1^ was associated with the benzene ring stretching of C=C. Quantitative measurement of the THC release from the hydrogels demonstrated the possible topical use of the PVA hydrogels to deliver THC continuously. A better-controlled release of THC was obtained with the help of PG, VG, or both, as compared to the PVA hydrogel without PG or VG. Finally, we demonstrated that the THC-rich hydrogels that contain PV, VG or both promoted skin fibroblast proliferation with a higher effect with the THC-rich PVA-PG-VG. Altogether, our results demonstrated the possible combination of PVA with PG and VG to generate useful THC-rich hydrogels for cannabinoid delivery. Because THC is lipophilic, our study suggests the possible delivery of THC when in topical contact with the tissues, including skin and oral mucosa, as the cells have lipid-rich membranes. Our THC-rich PVA-PG-VG hydrogels, therefore, may have the potential as a drug carrier for topical use to treat tissue inflammation.

## Data Availability

The original contributions presented in the study are included in the article/Supplementary material, further inquiries can be directed to the corresponding author.
